# INDUCE-2: A Phase I/II, open-label, two-part study of feladilimab in combination with tremelimumab in patients with advanced solid tumors

**DOI:** 10.1007/s00262-023-03623-z

**Published:** 2024-02-13

**Authors:** John F. Hilton, Patrick A. Ott, Aaron R. Hansen, Zujun Li, Matthen Mathew, Cristina H. Messina, Vimal Dave, Xiao Ji, Natalie O. Karpinich, Steven Hirschfeld, Marc Ballas, Dan P. Zandberg

**Affiliations:** 1 The Ottawa Hospital Cancer Centre, Ottawa, ON Canada; 2 Dana Farber Cancer Institute, Boston, MA USA; 3 Princess Margaret Cancer Centre, Toronto, ON Canada; 4 New York University, New York, NY USA; 5 Columbia University Irving Medical Center, New York, NY USA; 6 GSK, Collegeville, PA USA; 7 GSK, Bengaluru, Karnataka India; 8 UPMC Hillman Cancer Center, 5150 Centre Avenue, Pittsburgh, PA 15232 USA

**Keywords:** Immunotherapy, Biomarkers, Clinical-trial results, Tumor microenvironment, Immunology

## Abstract

Combining immunotherapies with distinct mechanisms of action has the potential to overcome treatment resistance and improve outcomes. The inducible T-cell co-stimulator (ICOS) agonist feladilimab is directed at enhancing T-cell activation and function, thereby promoting an antitumor response. INDUCE-2 (NCT03693612) was a Phase I/II, open-label, two-part study evaluating the anti-ICOS agonist feladilimab in combination with the anti-CTLA-4 antibody tremelimumab in patients with select advanced solid tumors. Objectives of Part 1 were to determine the safety, tolerability, and recommended phase 2 dose (RP2D) of feladilimab in combination with tremelimumab. In Part 2, the antitumor activity of the combination (administered at the RP2D determined in Part 1) was to be assessed in patients with relapsed/refractory head and neck squamous cell carcinoma. Primary endpoints included the rates of dose-limiting toxicities (DLTs), adverse events (AEs), AEs of special interest, and serious AEs. Secondary endpoints included overall response rate, while biomarker assessment was exploratory. A total of 26 patients were enrolled, 18 (69%) of whom had completed the study at end date. One patient, in the highest dose group (24/225 mg feladilimab/tremelimumab), experienced a DLT 18 days after the first dose of study treatment. All patients experienced at least one AE; AEs led to treatment discontinuation in four (15%) patients. Partial response was observed in one patient. Feladilimab in combination with tremelimumab was well-tolerated but showed limited efficacy. Based on the totality of data from Part 1, it was decided not to continue with Part 2.

**Supplementary Information:**

The online version contains supplementary material available at 10.1007/s00262-023-03623-z.

## Introduction

Immune checkpoint inhibitors have become an integral part of cancer therapy and are used to treat a variety of solid tumors, including head and neck squamous cell carcinoma (HNSCC), non-small cell lung cancer (NSCLC), and urothelial carcinoma (UC); however, many patients experience primary or secondary resistance [1–3]. Cancer cells can escape or develop resistance to host-mediated immunity and immune checkpoint inhibitors through multiple mechanisms, including the acquisition of mutations, loss or downregulation of antigen-processing machinery resulting in defective antigen presentation, suppression of immune reactions through induction of regulatory T cells (Tregs) and immunosuppressive cytokines, and activation of alternate immune checkpoint inhibitory pathways [4–7]. Due to these resistance mechanisms, immune checkpoint inhibitors as monotherapy are generally insufficient to elicit durable responses in most patients [1, 3]. Thus, there is a need for novel therapeutic targets and combinations to address these resistance mechanisms and achieve durable clinical benefit in a broader patient population.

Inducible T-cell co-stimulator (ICOS) is a co-stimulatory receptor highly expressed in tumor-infiltrating lymphocytes in many cancer types [8, 9]. ICOS signaling augments T-cell proliferation and directs memory cell development via cytokine production [8, 10, 11]. Suppression of cytotoxic T-lymphocyte-associated protein 4 (CTLA-4) upregulates ICOS expression on T cells [9]. Furthermore, frequencies of ICOS+ , CD4+ T cells were increased following anti-CTLA-4 therapy in patients with NSCLC, which was associated with improved response [12]. Therefore, the combination of an ICOS agonist and a CTLA-4 inhibitor may improve antitumor responses in patients with advanced solid tumors.

Feladilimab is a humanized ICOS agonist immunoglobulin G4 monoclonal antibody (mAb) that targets ICOS-expressing CD4+ and CD8+ effector T cells [13]. It was designed to enhance T-cell function and promote an antitumor response without depleting ICOS-expressing cells [13]. In the HNSCC expansion cohort of the first-in-human feladilimab study INDUCE-1 (NCT02723955), feladilimab monotherapy and combination therapy with pembrolizumab (a programmed death receptor protein 1 [PD-1]-targeting mAb) had a manageable safety profile and showed evidence of antitumor activity in patients with HNSCC who previously received anti-PD-1/programmed death-ligand 1 (PD-L1) treatment [14].

INDUCE-2 was a Phase I/II, open-label, two-part study (207871; NCT03693612) designed to investigate feladilimab in combination with the anti-CTLA-4 blocking monoclonal antibody tremelimumab. Part 1 of the study aimed to evaluate the safety, tolerability, and determine the recommended phase 2 dose (RP2D) of feladilimab in combination with tremelimumab in patients with select advanced solid tumors. Part 2 aimed to estimate overall survival (OS) in patients with PD-1-/PD-L1-experienced relapsed/refractory (RR) HNSCC treated with study intervention compared to those treated with investigator’s choice of standard of care (SOC).

## Methods

### Study design

INDUCE-2 was a Phase I/II, open-label study investigating feladilimab in combination with tremelimumab with a two-part design (Supplementary Fig. 1). Part 1 enrolled patients with advanced selected solid tumors in a dose-escalation design (described below). Part 2 was planned to investigate dose expansion in patients with RR HNSCC who had progressed after receiving at least one platinum-based chemotherapy and at least one anti-PD-1/PD-L1 therapy (whether in combination or given as monotherapy). The study comprised three periods: screening (assessments up to 30 days prior to first dose); treatment (until disease progression, unacceptable toxicity, or death); and follow-up.

### Study eligibility

Eligible patients for Part 1 were ≥ 18 years of age with a documented diagnosis of a locally advanced or metastatic relapsed solid tumor of cutaneous melanoma, HNSCC, NSCLC, UC, clear cell renal cancer, or castrate-resistant prostate cancer, who had progressed following SOC therapy. Selection of these tumors was based on evidence of their reported and/or predicted response to immune checkpoint therapies [15–20]. Patients were also required to have measurable disease as per the Response Evaluation Criteria in Solid Tumors version 1.1 (RECIST v.1.1), an Eastern Cooperative Oncology Group Performance Status of 0 or 1, adequate organ function, and consent to collection of tumor tissue samples [21, 22]. Key exclusion criteria included patients who had received prior anti-CTLA-4 or ICOS-directed therapies, patients who had received four or more lines of therapy, or those who had received systemic anticancer therapies within 30 days of first dose. The full list of exclusion criteria are as follows: patients with solid tumors other than those listed above; patients who had received prior anti-CTLA-4 or ICOS-directed therapies; patients who had received at least four lines of therapy, or who had received systemic anti-cancer therapies within 30 days of first dose; unresolved prior treatment-related toxicity; central nervous system metastasis; autoimmune disease or syndrome requiring systemic treatment within the past 2 years; systemic steroids or other immunosuppressive agents within 7 days; history of idiopathic pulmonary fibrosis or pneumonitis; history of severe hypersensitivity to monoclonal antibodies; history or evidence of cardiac abnormalities within 24 weeks of enrollment; current unstable liver or biliary disease; patients with an active infection requiring systemic therapy.

### Treatment

For Part 1, feladilimab/tremelimumab was administered at 8/75 mg; 24/75 mg; 8/225 mg; 80/75 mg; 24/225 mg; and 80/225 mg. Feladilimab doses were selected based on the safety and exposure data observed in the Phase I first-time-in-human study (INDUCE-1; NCT02723955), along with predicted target engagement based on in vitro potency and feladilimab exposure predicted from a population pharmacokinetic model [13, 14]. Tremelimumab doses were selected based on prior monotherapy and combination studies [23–25]. Dose escalation followed a zone-based approach guided by the bivariate Continuous Reassessment Method model until the maximum tolerated dose or maximum administered dose combination was determined (Supplementary Fig. 1). Feladilimab was administered once every 3 weeks (Q3W); tremelimumab was administered Q3W for 6 doses, and then every 12 weeks (Q12W) thereafter. Tremelimumab was administered intravenously (IV) over an initial 60-min infusion, followed by feladilimab over a 30-min IV infusion administered 1–2 h following the end of tremelimumab infusion. The RP2D determined in Part 1 of the study was planned to be carried forward to Part 2.

### Objectives and endpoints

The primary objective of Part 1 of the study was to determine the safety, tolerability, and RP2D of feladilimab in combination with tremelimumab. Endpoints reported here include the frequency and severity of dose-limiting toxicities (DLTs), adverse events (AEs), AEs of special interest (AESIs), serious AEs (SAEs) and DLTs/AEs/SAEs leading to dose modifications, delays, or withdrawals. Changes in laboratory parameters (for example, serum analytes including albumin, alkaline phosphatase (ALP), alanine transaminase (ALT), aspartate transaminase (AST), creatinine, and sodium), vital signs, and safety parameters were also assessed.

Secondary endpoints included investigator-assessed clinical activity of feladilimab in combination with tremelimumab, evaluated as best overall confirmed response per RECIST v1.1 and immune RECIST (iRECIST), and pharmacokinetic (PK) parameters of feladilimab in combination with tremelimumab [22]. Blood and tumor-based biomarkers were assessed as exploratory endpoints.

### Assessments

AEs were coded using Medical Dictionary for Regulatory Activities (MedDRA), grouped by system organ class, and graded by the Investigator according to the National Cancer Institute – Common Terminology Criteria for Adverse Events (NCI-CTCAE) v.5.0. Radiographic evaluations of the chest, abdomen, and pelvis were conducted according to RECIST v1.1 at 9 weeks after the first study dose, and then every 6 weeks until Week 52, and Q12W thereafter [22]. PK profiles of feladilimab and tremelimumab were measured from patient blood samples collected at protocol-defined points, and concentrations calculated using validated bioanalytical methodologies.

Whole blood samples were collected pre-dose on Weeks 1, 2, 4, 7, 13, and 25, and shipped for in-stream flow cytometry analysis for markers of T-cell activation and proliferation at Covance Central Laboratory. Serum samples were analyzed for pro-inflammatory cytokine induction using the MSD 10-plex panel (Q-Squared Laboratories). Paired tumor biopsy samples (defined as a pretreatment sample collected any time after the end of previous therapy and prior to the first dose of study intervention/SOC, and an on-treatment sample collected at Week 7) were analyzed by multiplex immunofluorescence using a custom 18-marker MultiOmyx panel (NeoGenomics Laboratories, CA, USA). Gene expression analysis was performed on paired tumor biopsies using the Nanostring PanCancer IO360 panel.

### Statistical methods

A maximum of 26 patients were enrolled for Part 1. All patients who received at least one dose of tremelimumab or feladilimab were included for the safety assessment summary. Efficacy data were initially planned to be analyzed using Kaplan–Meier analysis for time-to-event data; however, the OS and progression-free survival data were later only listed for clinical review purposes. PK parameters were presented as descriptive summary statistics. No statistical analyses were performed on blood- and tumor-based biomarker data due to small sample numbers.

## Results

### Study population

A total of 42 patients were screened, of which 26 were enrolled and allocated to feladilimab/tremelimumab 8/75 mg (n = 1), 24/75 mg (n = 1), 8/225 mg (n = 5), 80/75 mg (n = 3), 24/225 mg (n = 16), respectively (Supplementary Fig. 2). At study completion, 18 (69%) patients had completed the study per protocol and 8 (31%) patients had withdrawn from the study (Supplementary Fig. 2). Based on the totality of available data from Part 1, and not due to safety, the decision was made to stop the study following feladilimab/tremelimumab dosing at 24/225 mg and not to continue to dose level 80/225 mg, or to move into Part 2 of the study.

Most patients were male (n = 17, 65%) and White/Caucasian (n = 24; 92%), and the median age was 65 (range: 36–83) years (Table 1). At initial diagnosis, primary tumor types included NSCLC (n = 9; 35%), HNSCC (n = 8; 31%), melanoma (n = 5; 19%), and bladder cancer (n = 4; 15%). The most common histology was adenocarcinoma (n = 10; 38%) followed by squamous cell carcinoma (n = 9; 35%). The median time since initial diagnosis was 410.5 (range: 27–2019) days. All patients had received prior anti-cancer therapy: 88% (n = 23) had received immunotherapy; 77% (n = 20) chemotherapy; 65% (n = 17) radiotherapy; and 85% (n = 22) had undergone cancer-related surgery (Table 1). Most patients who received immunotherapy, chemotherapy, and/or radiotherapy had received one prior treatment regimen of that type, and most patients who had undergone cancer-related surgery had undergone three prior procedures.

**Table 1:** Patient demographics and baseline disease characteristics

	**Feladilimab mg/tremelimumab mg**					
**Population**	**8/75 (N = 1)**	**24/75 (N = 1)**	**8/225 (N = 5)**	**80/75 (N = 3)**	**24/225 (N = 16)**	**Total (N = 26)**
**Sex, n (%)**						
Female	1 (100)	1 (100)	3 (60)	0	4 (25)	9 (35)
Male	0	0	2 (40)	3 (100)	12 (75)	17 (65)
**Age (years), median (range)**^**a**^	51 (51–51)	51 (51–51)	62 (45–72)	66 (58–83)	71 (36–83)	65 (36–83)
**Race, n (%)**^**b**^						
White/Caucasian	1 (100)	1 (100)	5 (100)	3 (100)	14 (88)	24 (92)
Black/African American	0	0	0	0	1 (6)	1 (4)
**Primary tumor type, n (%)**^**c**^						
Bladder	0	0	0	1 (33)	3 (19)	4 (15)
HNSCC	0	0	1 (20)	0	7 (44)	8 (31)
Melanoma	1 (100)	1 (100)	2 (40)	0	1 (6)	5 (19)
NSCLC	0	0	2 (40)	2 (67)	5 (31)	9 (35)
**Time since initial diagnosis (days), median (range)**^**d**^	676 (676–676)	512 (512–512)	640 (175–2019)	251 (92–470)	372 (27–1545)	411 (27–2019)
**Prior therapies, n (%)**						
Immunotherapy	1 (100)	1 (100)	5 (100)	3 (100)	13 (81)	23 (88)
Chemotherapy	0	0	4 (80)	3 (100)	13 (81)	20 (77)
Radiotherapy	1 (100)	1 (100)	5 (100)	1 (33)	9 (56)	17 (65)
Surgery	1 (100)	1 (100)	4 (80)	2 (67)	14 (88)	22 (85)

^a^Age is imputed when full date of birth is not provided; ^b^one patient in the 24/225 mg dose group reported race as Multiple; ^c^histology at initial diagnosis reported as adenocarcinoma in 10 patients, squamous cell carcinoma in 9 patients, and other in 7 patients; ^d^time since diagnosis is defined as earliest treatment start date minus the initial diagnosis date plus 1 day

*HNSCC* head and neck squamous cell carcinoma, *NSCLC* non-small cell lung cancer

### Exposure and DLTs

Median time of exposure ranged from 6.21 to 18.29 weeks for feladilimab, and 6.14 to 15.29 weeks for tremelimumab. The number of infusions ranged from three to six for feladilimab, and from three to five for tremelimumab. One patient had a dose delay for both feladilimab and tremelimumab due to an AE (hypoxia) and no patients had a dose interruption or incomplete infusion. One patient in the highest dose group (feladilimab 24 mg/tremelimumab 225 mg), experienced a DLT 18 days after the first dose of study treatment (Grade 3 diarrhea considered possibility related to treatment). A flexible sigmoidoscopy on study day 23 revealed moderately congested, erythematous mucosa with mucosal prolapse. Due to concerns of immunotherapy-induced colitis, treatment was discontinued and after intervention the Grade 3 diarrhea resolved on Day 32.

### Safety

All patients experienced at least one AE and 19 (73%) patients experienced treatment-related (TR)-AEs (Table 2), the most common of which were diarrhea (n = 9; 35%) and fatigue (n = 8; 31%). SAEs occurred in 12 (46%) patients, of which two (8%) were considered to have experienced TR-SAEs (one case each of diarrhea and colitis). AEs (colitis, diarrhea, fatigue, acute kidney injury, and dyspnea) led to permanent discontinuation in four (15%) patients (Tables 2 and 3). One fatal SAE, considered related to disease progression, occurred during the study (Table 2). All deaths (n = 17; 65%) were attributed to disease under study, and most (n = 15; 88%) occurred over 30 days after the last dose of study treatment (data not shown).

**Table 2:** Overview of AEs

	**Feladilimab mg/tremelimumab mg**					
**Category, n (%)**	**8/75 (N = 1)**	**24/75 (N = 1)**	**8/225 (N = 5)**	**80/75 (N = 3)**	**24/225 (N = 16)**	**Total (N = 26)**
**Any AE**	1 (100)	1 (100)	5 (100)	3 (100)	16 (100)	26 (100)
TR-AEs^a^	1 (100)	0	4 (80)	2 (67)	12 (75)	19 (73)
AEs leading to permanent discontinuation of study treatment^b^	0	0	1 (20)	0	3 (19)	4 (15)
AEs leading to dose interruption/delay of study treatment	0	0	3 (60)	0	4 (25)	7 (27)
**Any SAE**	0	0	3 (60)	3 (100)	6 (38)	12 (46)
TR-SAEs^c^	0	0	0	0	2 (13)	2 (8)
SAEs leading to permanent discontinuation of study treatment	0	0	1 (20)	0	2 (13)	3 (12)
SAEs leading to dose reduction	0	0	0	0	0	0
SAEs leading to dose interruption/delay of study treatment	0	0	1 (20)	0	1 (6)	2 (8)
**Fatal SAEs**	0	0	0	0	1 (6)	1 (4)

For patients receiving combination therapy, “study treatment” refers to feladilimab and/or tremelimumab. A worst-case approach was taken for patients with missing relatedness data; events with missing relatedness were assumed to be treatment related. ^a^The two most common TR-AEs were diarrhea (35%) and fatigue (31%); ^b^progressive disease and AEs were reported in two patients. However, in the study treatment discontinuation form, the reason reported was progressive disease; ^c^TR-SAEs experienced were diarrhea and colitis

*AE* adverse event, *SAE* serious adverse event, *TR* treatment related

**Table 3:** AEs leading to permanent discontinuation and AESIs

	**Feladilimab mg/tremelimumab mg**					
**Preferred term, n (%)**	**8/75 (N = 1)**	**24/75 (N = 1)**	**8/225 (N = 5)**	**80/75 (N = 3)**	**24/225 (N = 16)**	**Total (N = 26)**
**AEs leading to permanent discontinuation**	0	0	1 (20)	0	3 (19)	4 (15)
Colitis	0	0	0	0	1 (6)	1 (4)
Diarrhea	0	0	0	0	1 (6)	1 (4)
Fatigue	0	0	0	0	1 (6)	1 (4)
Acute kidney injury	0	0	0	0	1 (6)	1 (4)
Dyspnea	0	0	1 (20)	0	0	1 (4)
**AESIs**	0	0	2 (40)	0	7 (44)	9 (25)
Colitis	0	0	1 (20)	0	1 (6)	2 (8)
Endocrinopathies	0	0	0	0	2 (13)	2 (8)
Hepatitis	0	0	1 (20)	0	0	1 (4)
Nephritis and renal function	0	0	0	0	2 (13)	2 (8)
Other immune-mediated AEs	0	0	0	0	1 (6)	1 (4)
Skin adverse reactions	0	0	2 (40)	0	3 (19)	5 (19)

Individual patients could experience ≥ 1 listed AE

*AE* adverse event, *AESI* adverse event of special interest

The most frequently reported AEs of any cause were fatigue, diarrhea, and nausea (Supplementary Table 1). Most patients experienced a Grade ≥ 3 AE (n = 15; 58%), the most frequently reported being fatigue, acute kidney injury, anemia, diarrhea, dyspnea, embolism, and hypoxia (data not shown). Grade ≥ 3 TR-AEs occurred in five (19%) patients, four of which received feladilimab 24 mg/tremelimumab 225 mg (Supplementary Table 2). Nine (35%) patients experienced at least one AESI, and of these events all five skin-related AESIs, both cases of colitis, one of two cases of nephritis and renal function, and the one case of hepatitis were considered treatment-related (Table 3).

### Efficacy

**Fig. 1:**
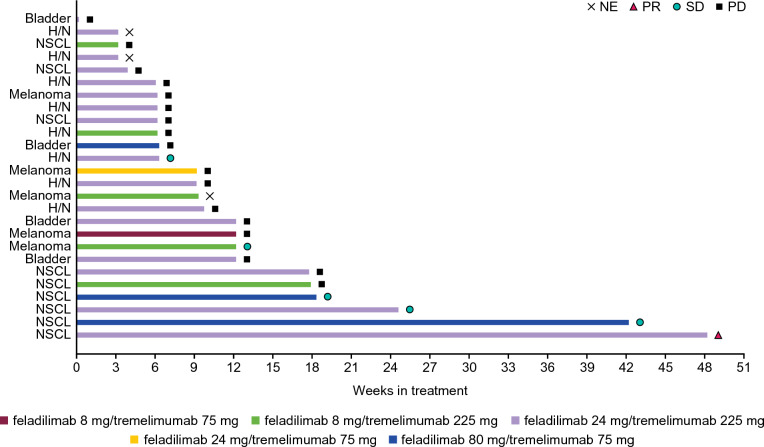
Best overall confirmed response for each patient. Plot of duration of study treatment (best overall confirmed response as per RECIST v1.1) for Part 1. Duration of treatment in weeks is defined as (last dosing date minus first dose date plus 1 day)/7 days. H/N head and neck, NE not evaluable, NSCL non-small cell lung, PD progressive disease, PR partial response, RECIST v1.1 Response Evaluation Criteria in Solid Tumors version 1.1, SD stable disease

One (4%) patient with NSCLC in the feladilimab 24 mg/tremelimumab 225 mg group achieved a confirmed partial response (PR), completed the study per protocol, and remained on study treatment for 337 days (Fig. 1). Stable disease (SD) was observed in five (19%) patients (including one patient with HNSCC, three patients with NSCLC, and one patient with melanoma). Of these patients, one was in the feladilimab/tremelimumab 8/225 mg group, two in the 80/75 mg group, and two in the 24/225 mg group (Fig. 1). Three patients (two in the 80/75 mg group and one in the 24/225 mg group) had SD ≥ 18 weeks.

Investigator-assessed best percent reduction from baseline in tumor measurement is shown in Supplementary Fig. 3a, and percent change in tumor measurement over time from baseline in Supplementary Fig. 3b.

### Pharmacokinetics

Median feladilimab plasma concentration–time data during Treatment Cycle 1 are shown in Supplementary Fig. 4 and Supplementary Table 3. Feladilimab exposure in combination with tremelimumab was dose proportional and generally consistent with expected patterns of accumulation for a mAb (Supplementary Table 3).

### Exploratory biomarkers

**Fig. 2:**
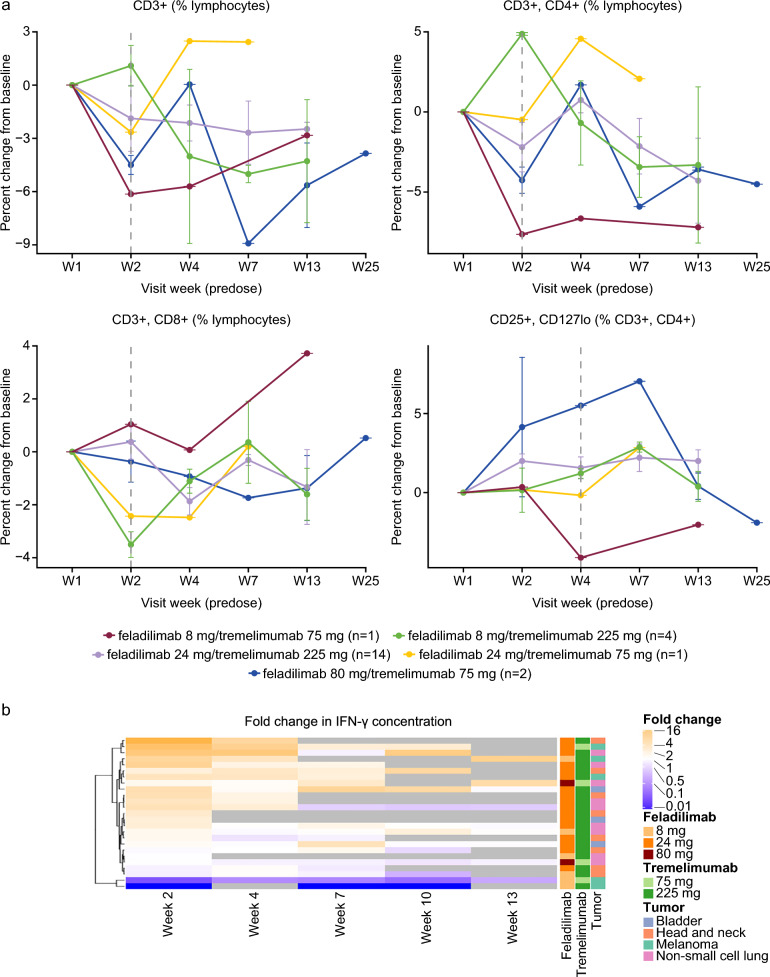
Pharmacodynamic analysis of blood-based biomarkers. Blood-based flow cytometry analysis showing percentage of CD3+ , CD4+ , CD8+ , and Tregs, across dose levels (**a**) and peripheral IFN-γ cytokine induction (**b**). Grey boxes in panel a represent no available data. No statistical analyses were performed due to small sample sizes per dose level. CD cluster of differentiation, IFN-γ interferon gamma, Tregs regulatory T cells, W week

The longitudinal assessment of peripheral immune cell populations showed a slight decrease in the percentage of CD3+ , CD4+ , and CD8+ T cells post-treatment across most dose levels (Fig. 2a). Similar trends were observed in pooled dose analyses showing the maximum pharmacodynamic changes for individual patients (Supplementary Fig. 5). Of the 10 evaluated cytokines from peripheral blood, only induction of interferon gamma (IFN-γ) was noted in most of the patients. At Week 2 (one week post-first dose), a ≥ twofold induction was observed in 11 out of 25 evaluated patients (Fig. 2b).

**Fig. 3:**
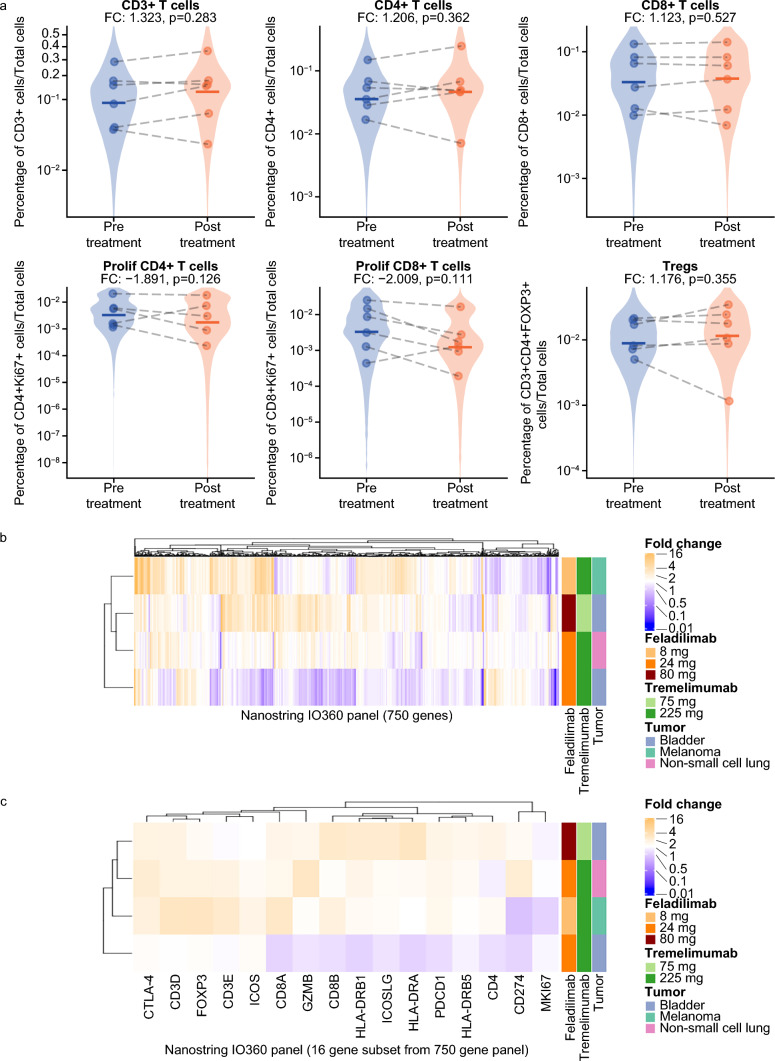
Assessment of T-cell populations and immune markers in pre- and post-treatment tumor biopsies. (**a**) Pooled dose analysis of multiplex immunofluorescence showing percentage of CD3+ , CD4+ , CD8+ , and Tregs in six baseline and on-treatment paired biopsies. (**b**) Fold-change gene expression analysis between baseline and Week 7 paired biopsies. (**c**) Fold-change gene expression analysis for a subset of genes related to ICOS biology and/or T-cell activation. CD cluster of differentiation, CTLA4 cytotoxic T-lymphocyte-associated antigen 4, FC fold change, FOXP3 forkhead box P3, GZMB granzyme B, HLA-DRA HLA class II histocompatibility antigen, DR alpha chain, HLA-DRB1, HLA class II histocompatibility antigen, DRB1 beta chain, HLA-DRB5 HLA class II histocompatibility antigen, DRB5 beta chain, ICOS inducible T-cell co-stimulator, ICOSLG ICOS ligand, MKI67 marker of proliferation Ki-67, PDCD1 programmed cell death protein-1, prolif proliferating, Tregs regulatory T cells

Multiplex immunofluorescence and gene expression analyses of paired tumor biopsies did not show any clear or consistent changes in immune cell markers. Pooled analysis of data from six paired tumor biopsies across several dose levels and tumor types did not show any statistically significant changes in T-cell populations or immune markers following combination treatment (Fig. 3a). Four paired biopsies were further evaluated for gene expression changes and there was minimal consistency in their gene expression profiles after combination treatment. Additional analyses focusing on a subset of genes associated with ICOS biology and/or T-cell activation showed no overlap in genes exhibiting a ≥ twofold increase in gene expression among all four patients (Fig. 3b, c).

**Fig. 4:**
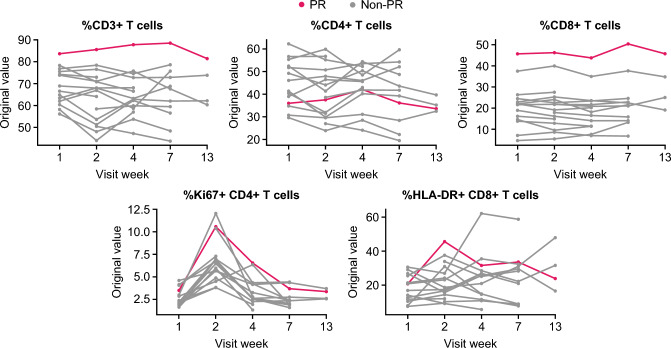
Flow cytometry analysis of the PR patient receiving feladilimab 24 mg/tremelimumab 255 mg. Blood-based flow cytometry analysis showing percentage of CD3+ , CD4+ , CD8+ , Ki67/CD4+ and HLA-DR+ /CD8+ T cells across time. Red line indicates T-cell frequencies in the patient achieving a PR relative to other patients (gray lines) enrolled in the same dose-level cohort. CD cluster of differentiation, HLA-DR human leukocyte antigen DR isotype, Ki67 marker of proliferation Ki-67, PR partial response

Among the 26 patients enrolled, a PR was achieved in one patient with study treatment (24 mg feladilimab plus 225 mg tremelimumab). Flow cytometry analysis showed that this responder displayed the highest levels of CD3+ and CD8+ T-cells relative to other patients enrolled in the same dose-level cohort (Fig. 4, red line). Further, this patient had a marked increase in the percentage of proliferating (Ki67+ /CD4+) and activated (HLA-DR+ CD8+) T-cells at Week 2 (one week post first dose).

## Discussion

The INDUCE-2 study aimed to evaluate the safety, tolerability, RP2D, and efficacy of feladilimab (an anti-ICOS agonist) administered in combination with tremelimumab (an anti-CTLA-4 monoclonal antibody) over two parts. Part 1, which included advanced solid tumors, revealed that the overall safety profile of feladilimab at each dose (8 mg, 24 mg, and 80 mg) in combination with tremelimumab (75 mg and 225 mg) was similar to the respective safety profiles of each as a monotherapy. The doses tested were well tolerated, and AEs were consistent with known toxicities. No new toxicities emerged related to either therapy, qualitatively or quantitatively. Feladilimab exposure in combination with tremelimumab was comparable to feladilimab exposure when administered at equivalent body weight-based doses as monotherapy. Overall, feladilimab/tremelimumab combination was found to be safe and well tolerated at the highest tested dose of 24/225 mg.

The efficacy of feladilimab in combination with tremelimumab was modest at all doses studied, with treatment achieving PR in only one patient, and SD in five patients. While this combination of feladilimab and tremelimumab is not being further developed, biomarker-based biological activity was observed, including in the one patient with NSCLC who experienced a PR and remained on study treatment for almost one year.

Interpretation of the biomarker data is challenged by the relatively small number of patients assessed across five dose levels and lack of clear trends in cell frequencies and cytokine levels between patients and over time; therefore, no clear conclusions can be drawn. In contrast to our findings, a previous study investigating tremelimumab monotherapy in patients with advanced, unresectable mesothelioma found a significant increase in blood CD3+ and CD4+ T-cells following treatment [26]. However, the monotherapy study used a higher dose of tremelimumab compared to the present study, and further investigation is required before conclusions can be made [26].

INDUCE-2 demonstrated the feasibility of combining an ICOS agonist with a CTLA-4 inhibitor. Furthermore, the zone-based dose-escalation design was feasible. The assessment of immune cell populations and other immune markers, while inconclusive, demonstrate the feasibility and importance of integrating correlative analyses into early-phase clinical studies. This allows for the potential to develop an increased understanding of drug mechanism of action(s), as well as potential predictive and prognostic exploration to identify patients who may benefit from specific immunotherapy combinations.

In conclusion, the INDUCE-2 study demonstrated that feladilimab in combination with tremelimumab is well tolerated but had limited efficacy in patients with select advanced solid tumors. Overall, this study design was able to conclude, with sufficient quality, study questions related to the primary and secondary endpoints of the protocol, as well as demonstrate the feasibility of a zone-based dose-escalation approach and integration of correlative biomarker assessments in early-phase clinical studies.

### Supplementary Information

Below is the link to the electronic supplementary material.

Supplementary file1 (DOCX 567 KB)

## Abbreviations

AEs: Adverse events
AESIs: AEs of special interest
ALP: Alkaline phosphatase
ALT: Alanine transaminase
AST: Aspartate transaminase
CTLA-4: Cytotoxic T-lymphocyte-associated protein 4
DLTs: Dose-limiting toxicities
HNSCC: Head and neck squamous cell carcinoma
ICOS: Inducible T-cell co-stimulator
iRECIST: Immune RECIST
IV: Intravenous
mAb: Monoclonal antibody
MedDRA: Medical Dictionary for Regulatory Activities
NCI-CTCAE: National Cancer Institute – Common Terminology Criteria for Adverse Events
NSCLC: Non-small cell lung cancer
OS: Overall survival
PD-1: Programmed death receptor protein 1
PD-L1: Programmed death-ligand 1
PK: Pharmacokinetic
PR: Partial response
Q3W: Once every 3 weeks
Q12W: Every 12 weeks
RECIST v.1.1: Response Evaluation Criteria in Solid Tumors version 1.1
RP2D: Recommended phase 2 dose
RR: Relapsed/refractory
SAEs: Serious AEs
SD: Stable disease
SOC: Standard of care
TR: Treatment-related
Tregs: Regulatory T cells
UC: Urothelial carcinoma

## Declarations

### Data availability statement

GSK makes available anonymized individual participant data and associated documents from interventional clinical studies that evaluate medicines, upon approval of proposals submitted to: https://www.gsk-studyregister.com/en/

## References

[1] Ferris RL, Blumenschein G, Fayette J (2016). Nivolumab for recurrent squamous-cell carcinoma of the head and neck. N Engl J Med.

[2] National Comprehensive Cancer Network (2022) NCCN clinical practice guidelines in oncology – bladder cancer. https://www.nccn.org/guidelines/guidelines-detail?category=1&id=1417. Accessed May 2022

[3] Seidel JA, Otsuka A, Kabashima K (2018). Anti-PD-1 and anti-CTLA-4 therapies in cancer: mechanisms of action, efficacy, and limitations. Front Oncol.

[4] Koyama S, Akbay EA, Li YY (2016). Adaptive resistance to therapeutic PD-1 blockade is associated with upregulation of alternative immune checkpoints. Nat Commun.

[5] Johnsen AK, Templeton DJ, Sy M-S, Harding CV (1999). Deficiency of transporter for antigen presentation (TAP) in tumor cells allows evasion of immune surveillance and increases tumorigenesis. J Immun.

[6] Yokokawa J, Cereda V, Remondo C, Gulley JL, Arlen PM, Schlom J, Tsang KY (2008). Enhanced functionality of CD4+CD25(high)FoxP3+ regulatory T cells in the peripheral blood of patients with prostate cancer. Clin Cancer Res.

[7] Zaretsky JM, Garcia-Diaz A, Shin DS (2016). Mutations associated with acquired resistance to PD-1 blockade in melanoma. NEJM.

[8] Hutloff A, Dittrich AM, Beier KC, Eljaschewitsch B, Kraft R, Anagnostopoulos I, Kroczek RA (1999). ICOS is an inducible T-cell co-stimulator structurally and functionally related to CD28. Nature.

[9] Fan X, Quezada SA, Sepulveda MA, Sharma P, Allison JP (2014). Engagement of the ICOS pathway markedly enhances efficacy of CTLA-4 blockade in cancer immunotherapy. J Exp Med.

[10] Schepp J, Chou J, Skrabl-Baumgartner A (2017). 14 Years after discovery: clinical follow-up on 15 patients with inducible co-stimulator deficiency. Front Immunol.

[11] Soldevilla MM, Villanueva H, Meraviglia-Crivelli D (2019). ICOS costimulation at the tumor site in combination with CTLA-4 blockade therapy elicits strong tumor immunity. Mol Ther.

[12] Yi JS, Ready N, Healy P (2017). Immune activation in early-stage non–small cell lung cancer patients receiving neoadjuvant chemotherapy plus ipilimumab. Clin Cancer Res.

[13] Balar AV, Moreno V, Angevin E (2021). Inducible T-cell co-stimulatory (ICOS) receptor agonist, feladilimab (fela), alone and in combination (combo) with pembrolizumab (P): Results from INDUCE-1 urothelial carcinoma (UC) expansion cohorts (ECs). Clin Oncol.

[14] Angevin E, Groenland SL, Lim AML (2020). Updated analysis of the inducible T-cell co-stimulatory receptor (ICOS) agonist, GSK3359609 (GSK609), combination with pembrolizumab (PE) in patients (pts) with anti-PD-1/L1 treatment-naïve head and neck squamous cell carcinoma (HNSCC). Clin Oncol.

[15] Hodi FS, O’Day SJ, McDermott DF (2010). Improved survival with ipilimumab in patients with metastatic melanoma. NEJM.

[16] Seiwert TY, Burtness B, Mehra R (2016). Safety and clinical activity of pembrolizumab for treatment of recurrent or metastatic squamous cell carcinoma of the head and neck (KEYNOTE-012): an open-label, multicentre, phase 1b trial. Lancet Oncol.

[17] Gettinger SN, Shepherd FA, Antonia SJ (2014). First-line nivolumab (anti-PD-1; BMS-936558, ONO-4538) monotherapy in advanced NSCLC: safety, efficacy, and correlation of outcomes with PD-L1 status. Clin Oncol.

[18] Powles T, Eder JP, Fine GD (2014). MPDL3280A (anti-PD-L1) treatment leads to clinical activity in metastatic bladder cancer. Nature.

[19] Vano YA, Elaidi R, Bennamoun M (2022). Nivolumab, nivolumab-ipilimumab, and VEGFR-tyrosine kinase inhibitors as first-line treatment for metastatic clear-cell renal cell carcinoma (BIONIKK): a biomarker-driven, open-label, non-comparative, randomised, phase 2 trial. Lancet Oncol.

[20] Slovin SF, Higano CS, Hamid O (2013). Ipilimumab alone or in combination with radiotherapy in metastatic castration-resistant prostate cancer: results from an open-label, multicenter phase I/II study. Ann Oncol.

[21] Oken MM, Creech RH, Tormey DC, Horton J, Davis TE, McFadden ET, Carbone PP (1982). Toxicity and response criteria of the eastern cooperative oncology group. Am J Clin Oncol.

[22] Eisenhauer EA, Therasse P, Bogaerts J (2009). New response evaluation criteria in solid tumours: revised RECIST guideline (version 1.1). Eur J Cancer.

[23] AstraZeneca (2018) A Phase III randomized, open-label, multi-center, global study of MEDI4736 in combination with tremelimumab therapy or MEDI4736 monotherapy versus standard of care platinum-based chemotherapy in first-line treatment of patients with Advanced or Metastatic Non-SmallCell Lung Cancer (NSCLC) (MYSTIC). https://classic.clinicaltrials.gov/ProvidedDocs/82/NCT02453282/Prot_006.pdf. Accessed October 2023

[24] Riudavets M, Naigeon M, Texier M (2022). Gefitinib plus tremelimumab combination in refractory non-small cell lung cancer patients harbouring EGFR mutations: The GEFTREM phase I trial. Lung Cancer.

[25] Vicier C, Isambert N, Cropet C, Hamimed M, Osanno L, Legrand F, de La Motte RT, Ciccolini J, Gonçalves A (2022). MOVIE: a phase I, open-label, multicenter study to evaluate the safety and tolerability of metronomic vinorelbine combined with durvalumab plus tremelimumab in patients with advanced solid tumors. ESMO Open.

[26] Calabrò L, Morra A, Fonsatti E (2015). Efficacy and safety of an intensified schedule of tremelimumab for chemotherapy-resistant malignant mesothelioma: an open-label, single-arm, phase 2 study. Lancet Respir Med.

