# Supplementary material for: INDUCE-2: A Phase I/II, open-label, two-part study of feladilimab in combination with tremelimumab in patients with advanced solid tumors
Source: Cancer Immunol Immunother. 2024 Feb 13;73(3):44. doi: 10.1007/s00262-023-03623-z (PMC10864474; doi:10.1007/s00262-023-03623-z)
Supplement: Supplementary file 1 — Supplementary file1 (DOCX 567 KB) [file 262_2023_3623_MOESM1_ESM.docx]

Supplementary Materials

Supplementary Table 1 Summary of common AEs (≥10% in total population; any cause)

|  | **Feladilimab mg/tremelimumab mg** | | | | | |
| --- | --- | --- | --- | --- | --- | --- |
| **Preferred term, n (%)** | **8/75  (N=1)** | **24/75 (N=1)** | **8/225 (N=5)** | **80/75 (N=3)** | **24/225 (N=16)** | **Total (N=26)** |
| Fatigue | 0 | 0 | 1 (20) | 1 (33) | 10 (63) | 12 (46) |
| Diarrhea | 1 (100) | 0 | 1 (20) | 0 | 8 (50) | 10 (38) |
| Nausea | 1 (100) | 0 | 1 (20) | 1 (33) | 6 (38) | 9 (35) |
| Anemia | 0 | 0 | 2 (40) | 1 (33) | 5 (31) | 8 (31) |
| Decreased appetite | 0 | 0 | 1 (20) | 1 (33) | 6 (38) | 8 (31) |
| Dyspnea | 0 | 0 | 3 (60) | 2 (67) | 2 (13) | 7 (27) |
| Hypoalbuminemia | 0 | 0 | 1 (20) | 1 (33) | 5 (31) | 7 (27) |
| Hyponatremia | 0 | 0 | 1 (20) | 1 (33) | 4 (25) | 6 (23) |
| Constipation | 0 | 0 | 1 (20) | 1 (33) | 3 (19) | 5 (19) |
| Vomiting | 0 | 0 | 1 (20) | 0 | 4 (25) | 5 (19) |
| Abdominal pain | 0 | 0 | 0 | 0 | 4 (25) | 4 (15) |
| Blood ALP increased | 0 | 0 | 1 (20) | 1 (33) | 2 (13) | 4 (15) |
| Blood creatinine increased | 0 | 0 | 1 (20) | 1 (33) | 2 (13) | 4 (15) |
| Edema peripheral | 0 | 0 | 1 (20) | 0 | 3 (19) | 4 (15) |
| ALT increased | 0 | 0 | 1 (20) | 0 | 2 (13) | 3 (12) |
| AST increased | 0 | 0 | 0 | 0 | 3 (19) | 3 (12) |
| Cough | 0 | 0 | 1 (20) | 0 | 2 (13) | 3 (12) |
| Dizziness | 0 | 0 | 0 | 0 | 3 (19) | 3 (12) |
| Embolism | 0 | 0 | 1 (20) | 1 (33) | 1 (6) | 3 (12) |
| Hypoxia | 0 | 0 | 1 (20) | 1 (33) | 1 (6) | 3 (12) |
| Pruritus | 0 | 0 | 1 (20) | 0 | 2 (13) | 3 (12) |

Individual patients could experience ≥1 listed AE.

AE, adverse event; ALP, alkaline phosphatase; ALT, alanine transaminase; AST, aspartate transaminase.

Supplementary Table 2 Summary of common Grade ≥3 TR-AEs (≥5% in total population)

|  | **Feladilimab mg/tremelimumab mg** | | | | | |
| --- | --- | --- | --- | --- | --- | --- |
| **Preferred term, n (%)** | **8/75  (N=1)** | **24/75 (N=1)** | **8/225 (N=5)** | **80/75 (N=3)** | **24/225 (N=16)** | **Total (N=26)** |
| **Any event** | 0 | 0 | 1 (20) | 0 | 4 (25) | 5 (19) |
| Acute kidney injury | 0 | 0 | 0 | 0 | 1 (6) | 1 (4) |
| AST increased | 0 | 0 | 0 | 0 | 1 (6) | 1 (4) |
| Colitis | 0 | 0 | 0 | 0 | 1 (6) | 1 (4) |
| Diarrhea | 0 | 0 | 0 | 0 | 1 (6) | 1 (4) |
| Fatigue | 0 | 0 | 0 | 0 | 1 (6) | 1 (4) |
| Hypertension | 0 | 0 | 1 (20) | 0 | 0 | 1 (4) |

Individual patients could experience ≥1 listed AE.

AST, aspartate transaminase; TR-AE, treatment-related adverse event.

Supplementary Table 3 Summary of select feladilimab plasma PK parameters from Treatment Cycle 1 and Cycle 6 dosing

|  | **Feladilimab mg/tremelimumab mg** | | | | |
| --- | --- | --- | --- | --- | --- |
| **PK parameter** | **8/75 (N=1)** | **24/75 (N=1)** | **8/225 (N=5)** | **80/75 (N=3)** | **24/225 (N=16)** |
| C_max_ (ng/mL)  (95% CI) [CVb%] | 2758.0 | 5965.0 | 2686.6  (1961.5, 3679.6)  [25.7%] | 28340.9  (9440.5, 85080.3)  [12.3%] | 6098.3  (5340.5, 6963.7)  [25.3%] |
| T_max_ (h), median (range^a^) | 0.5  (1–1) | 0.5  (1–1) | 0.6  (1–5) | 0.5  (1–1) | 0.7  (1–5) |
| C_min_ Cycle 1 (ng/mL)  (95% CI) [CVb%] | 462.0 | ND | 348.0  (245.0, 494.2)  [14.2%] | 3197.0  (1507.3, 6780.5)  [31.0%] | 856.4  (700.4, 1047.1)  [35.9%] |
| C_min_ Cycle 6 (ng/mL) (95% CI) [CVb%] | ND | ND | 301.0 | 6922.5  (28.9, 1657524.6)  [67.1%] | 1546.7  (90.4, 26452.5)  [32.4%] |
| AUC (0-t) (h*ng/mL)  (95% CI) [CVb%] | 543733.0 | 615019.6 | 337344.6  (206989.9, 549792.0)  [40.9%] | 4622881.0  (1140376.9, 18740320.8)  [15.7%] | 1066361.5  (907205.7, 1253438.8)  [31.0%] |
| AUC (0-τ) (h*ng/mL)  (95% CI) [CVb%] | 545097.4 | ND | 453561.5  (159692.2, 1288215.6)  [11.7%] | 4552653.1  (878777.3, 23585783.4)  [18.5%] | 1055659.5  (851851.6, 1308229.0)  [23.5%] |

Data are represented as geometric means unless otherwise stated. AUC_(0-t)_ from time zero to the last quantifiable concentration. AUC_(0-τ)_ is from time zero to pre-dose of the next dosing period at Week 4 (Cycle 2, Day 1). Each Cycle refers to the three-week phase from receiving one dose to just prior to receiving the next dose. ^a^Values for the range have been rounded to whole numbers.

AUC, area under the curve; CI, confidence interval; C_max_, maximum serum concentration; C_min_, minimum serum concentration; CVb, between subject coefficient of variation; ND, not done; PK, pharmacokinetics; T_max_, time to peak concentration.

Supplementary Fig. 1 Study design


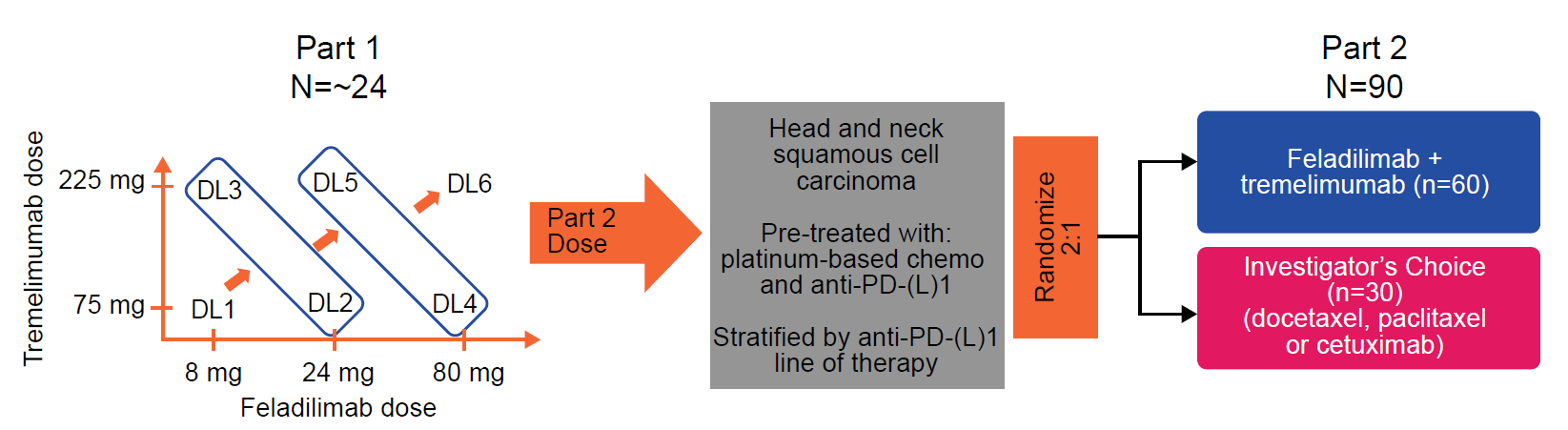


DL, dose level; PD-L1, programmed death-ligand 1.

Supplementary Fig. 2 Patient disposition


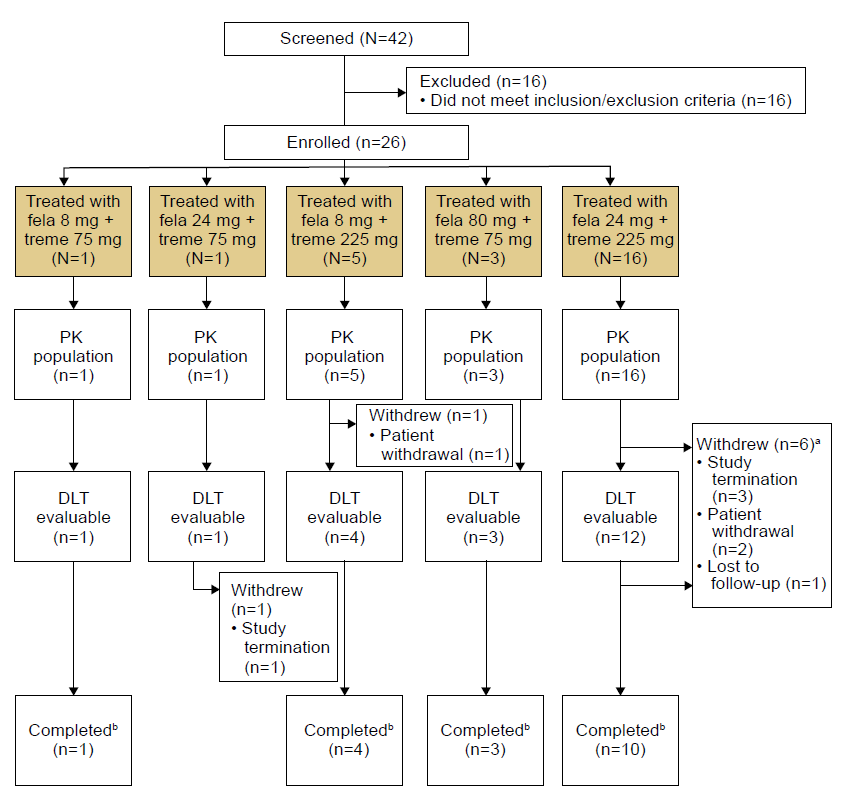


^a^Patients may have more than one reason for withdrawal; ^b^Patients were considered to have completed the study if they: (i) have completed screening assessments, and have received at least two doses of study intervention or have received one dose but have experienced a DLT and were observed during the 28-day DLT observation period, and the follow-up visit for safety (if required); or (ii) have completed screening assessments and have died while receiving study intervention or during the follow-up period for safety.

DLT, dose-limiting toxicity; fela, feladilimab; PK, pharmacokinetics; treme, tremelimumab.

Supplementary Fig. 3 Investigator-assessed best percent reduction from baseline in tumor measurement


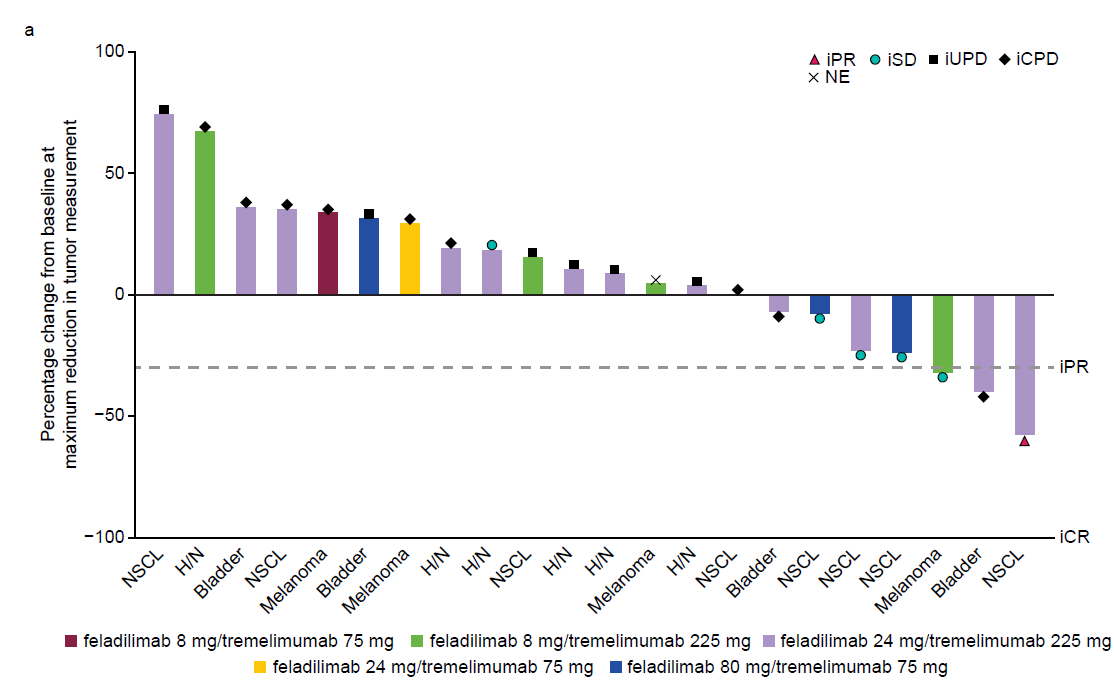


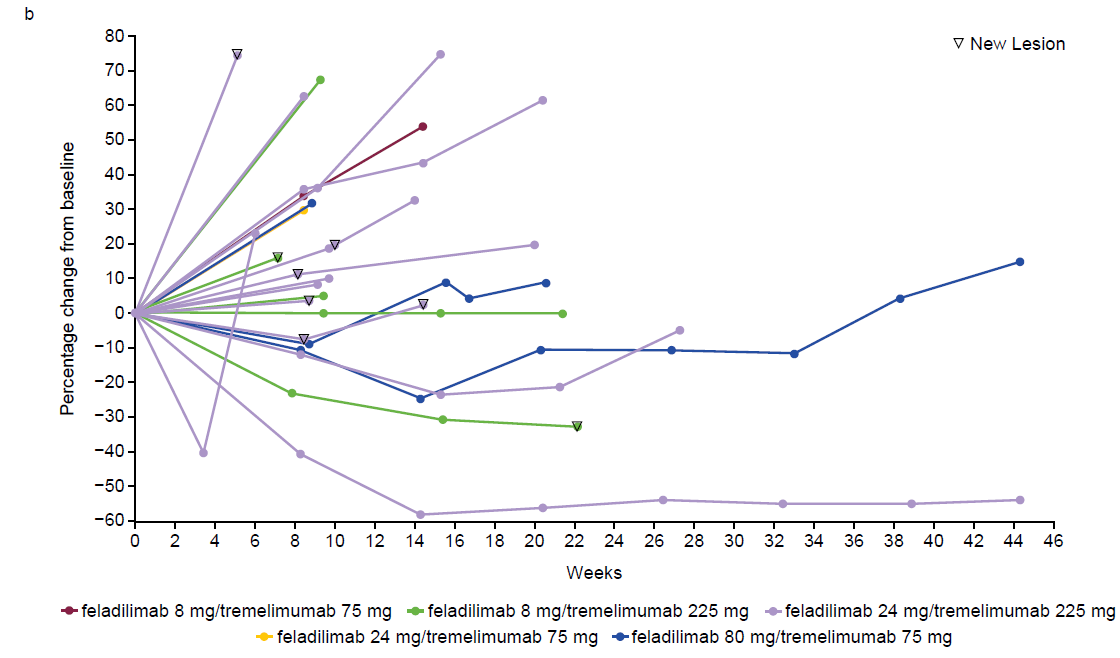


a) Investigator-assessed best percent reduction from baseline in tumor measurement (iRECIST). b) Percent change over time from baseline in tumor measurement.

CPD, confirmed progressive disease; CR, complete response; H/N, head and neck; i, per iRECIST; iRECIST, Immune Response Evaluation Criteria in Solid Tumors; NE, not evaluable; NSCL, non-small cell lung; PD, progressive disease; PR, partial response; SD, stable disease; UPD, unconfirmed progressive disease.

Supplementary Fig. 4 Median feladilimab plasma concentration–time plot during Cycle 1 dosing by dose level


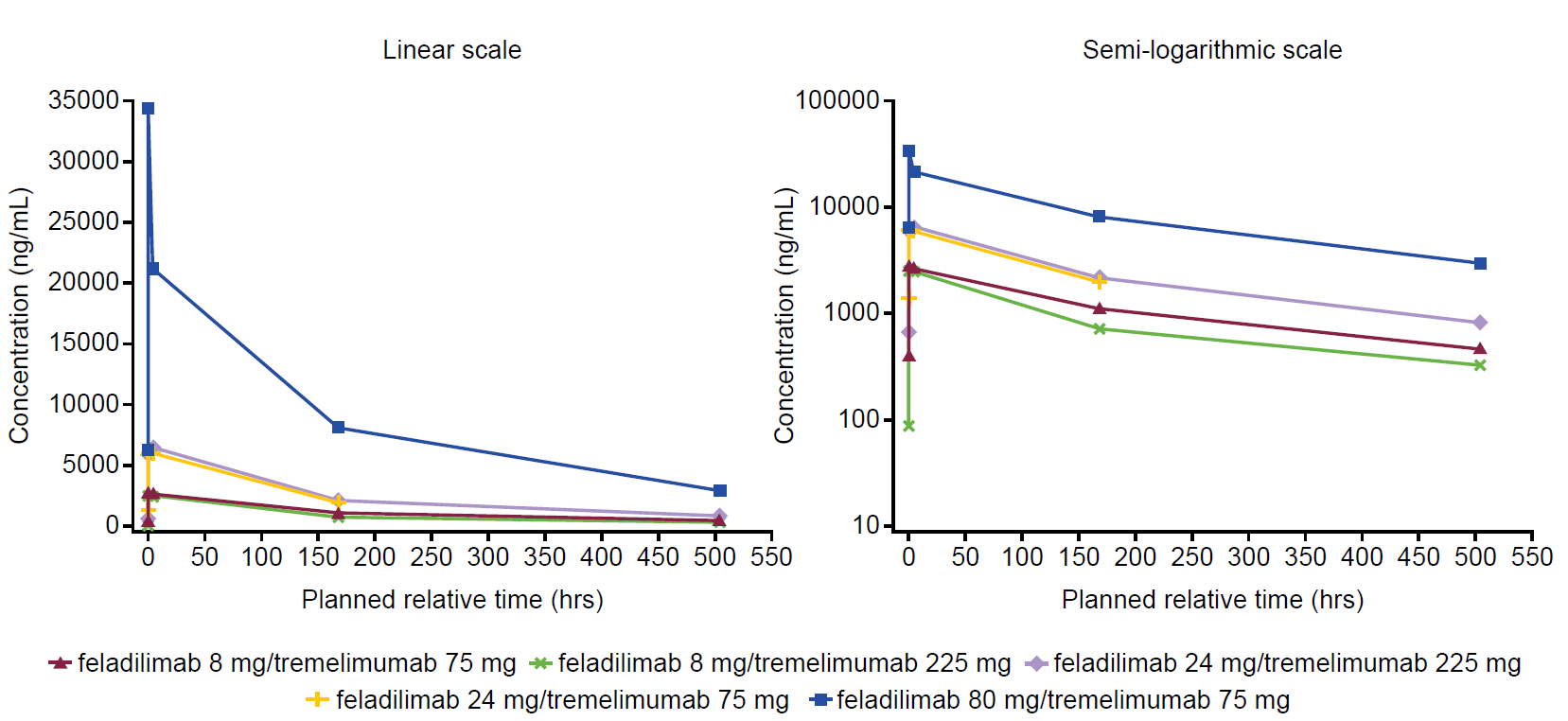


Each Cycle refers to the three-week phase from receiving one dose to just prior to receiving the next dose.

## Supplementary Fig. 5 Maximal pharmacodynamic changes for peripheral T-cell populations


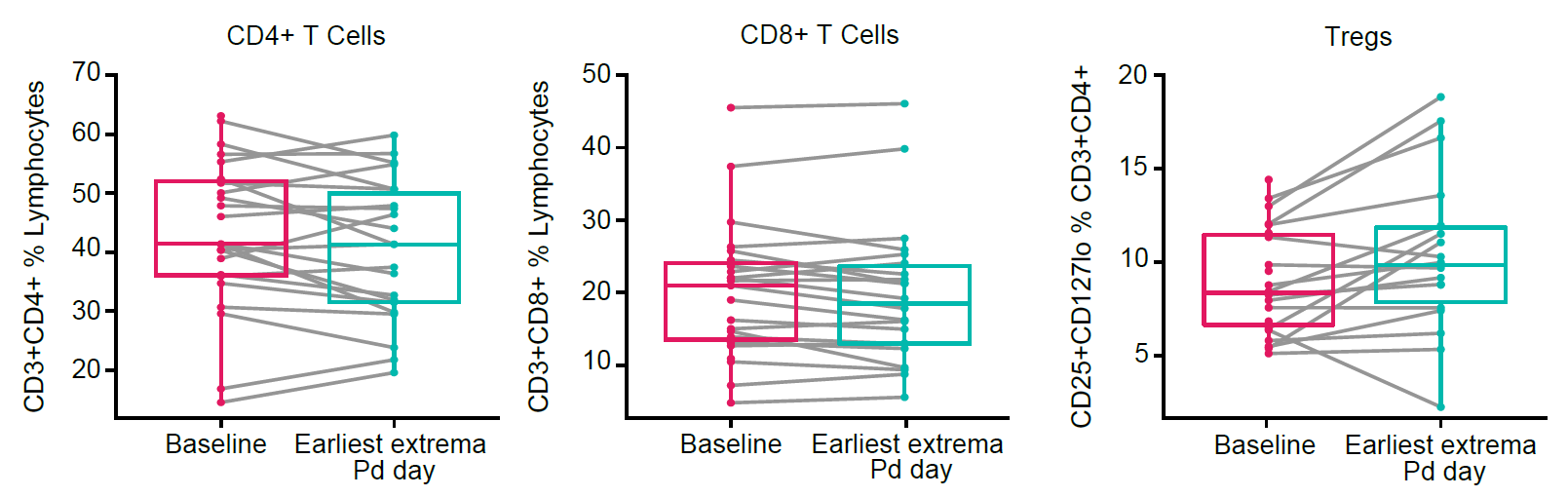


Blood-based flow cytometry analysis of individual patients from all dose levels showing percentages of CD4+, CD8+, and Tregs at baseline and the earliest post-dose time point with maximal Pd effect.

CD, cluster of differentiation; Pd, pharmacodynamic; Tregs, regulatory T cells.
